# Triaxiality of neutron-rich ruthenium nuclei studied by lifetime measurements

**DOI:** 10.1140/epja/s10050-025-01782-4

**Published:** 2026-03-02

**Authors:** J. S. Heines, V. Modamio, A. Görgen, W. Korten, E. Clément, J. Dudouet, A. Lemasson, J. Ljungvall, J. M. Allmond, T. R. Rodríguez, A. M. Bruce, D. T. Doherty, A. Esmaylzadeh, E. R. Gamba, J. Gerl, G. Georgiev, L. Knafla, P. Koseoglou, S. Lalkovski, H. -J. Li, G. Pasqualato, L. G. Pedersen, S. Pietri, D. Ralet, E. Sahin, S. Siem, P. -A. Söderström, C. Theisen, T. Tornyi

**Affiliations:** 1 Department of Physics, University of Oslo, Oslo, Norway; 2 Norwegian Nuclear Research Centre, Oslo, Norway; 3 IRFU, CEA, Université Paris-Saclay, Gif-sur-Yvette, France; 4 Grand Accélérateur National d’Ions Lourds, CEA/DRF-CNRS/IN2P3, Boulevard Henri Becquerel, 14076 Caen, France; 5 Université Claude Bernard Lyon-1, CNRS/IN2P3, UMR 5822, IP2I, Villeurbanne, France; 6 Université Paris-Saclay, IJCLab, IN2P3/CNRS, Orasy, France; 7 Université de Strasbourg, CNRS, IPHC, UMR 7178, Strasbourg, France; 8 Physics Division, Oak Ridge National Laboratory, Oak Ridge, TN 37831 USA; 9 Departamento de Física Atómica, Molecular y Nuclear, Universidad de Sevilla, Sevilla, Spain; 10 School of Computing, Engineering and Mathematics, Brighton University, Brighton, UK; 11 Department of Physics, University of Surrey, Guildford, UK; 12 Institut für Kernphysik, Universität zu Köln, Köln, Germany; 13 GSI Helmholtzzentrum für Schwerionenforschung GmbH, Darmstadt, Germany; 14 Faculty of Physics, Sofia University ’St. Kliment Ohridski’, 1164 Sofia, Bulgaria; 15 Department of Physics, Institute for Nuclear Physics, TU Darmstadt, Darmstadt, Germany; 16 Extreme Light Infrastructure (ELI-NP), Horia Hulubei National Institute for R&D in Physics and Nuclear Engineering (IFIN-HH), Str. Reactorului No. 30, 077125 Bucharest-Măgurele, Romania; 17 HUN-REN Institute for Nuclear Research (ATOMKI), Debrecen, Hungary

## Abstract

The breaking of axial symmetry in nuclei enables otherwise precluded behaviours, making it an interesting phenomenon to study. Experimental fingerprints such as very low-lying $2_2^+$ states suggest pronounced triaxial deformation for the neutron-rich ruthenium isotopes. Nevertheless, theoretical calculations differ in the description of the triaxial deformation and its evolution with neutron number, making experimental data crucial to understanding it. We investigated the evolution of the degree of triaxiality and $\gamma$ rigidity in neutron-rich ruthenium isotopes by measuring lifetimes of excited states in $^{108-112}$Ru with the recoil distance Doppler-shift method. The experiment was carried out at the Grand Accélérateur National d’Ions Lourds using the Advanced Gamma Tracking Array coupled to the Variable Mode Spectrometer. We obtained *B*(*E*2) values for 29 transitions in the studied nuclei and compared them with fully microscopic symmetry conserving configuration mixing calculations, and phenomenological generalized triaxial rotor and triaxial particle-rotor models. The models generally reproduce the measured transition strengths, and show an increase in triaxiality with neutron number, reaching near maximum triaxiality in $^{112}$Ru. The results are consistent with a transition from $\gamma$ soft to $\gamma$ rigid motion as the neutron number increases.

## Introduction

The region of the nuclear chart between Sr ($Z=38$) and Ru ($Z=44$) with mass numbers around 100 to 110 is known for a wide range of interesting phenomena related to nuclear deformation. A very rapid onset of quadrupole deformation for the Sr and Zr isotopes at neutron number $N=60$ is manifested by a sudden drop in the energy of the first $2^+$ state and a sudden increase in the $B(E2;0^+\rightarrow 2^+)$ value and was explained by the interaction between the $\nu g_{7/2}$ and $\pi g_{9/2}$ spin-orbit partners [1] and the occupation of the shape-driving $\pi g_{9/2}$ and $\nu h_{11/2}$ orbitals [2]. The competition between near-spherical and well-deformed prolate shapes leads to shape coexistence in the transitional region with low-lying excited $0^+$ states, large $\rho (E0)$ values, and mixing between the spherical and deformed configurations [3–5]. The shape transition is much more gradual for the Mo and Ru isotopes, with evidence for shape coexistence in the Mo isotopes, but not in the Ru isotopes [6]. Along with the more gradual onset of deformation for these isotopes, there is evidence of an increasing deviation from axial symmetry. A recent theoretical work based on configuration interaction proposed new mechanisms explaining the origins of small and moderate triaxial deformations in heavy nuclei, and identified medium-mass nuclei with large triaxiality as cases of particular interest for further investigation [7].

The neutron rich ruthenium isotopes are ideal candidates for investigating large triaxial deformation. Indeed, they are considered to be some of the best examples of nuclei that exhibit triaxial deformation near the ground state in the entire nuclear chart. Calculations within the finite-range liquid drop model that compared ground-state energies for axially symmetric and triaxial deformation predict nuclei around $^{108}$Ru to have the largest gain in stability from triaxiality [8]. Triaxial deformation for the neutron-rich Ru isotopes is predicted by a wide range of theoretical calculations including Nilsson-Strutinsky calculations with a cranked Woods-Saxon potential [9], cranked shell model calculations [10], effective field theory [11], relativistic Hartree-Bogoliubov calculations [12], and Hartree-Fock-Bogoliubov calculations with Skyrme [13], Gogny [14, 15], and relativistic [16] energy density functionals. Most models predict an increase in $\gamma$ deformation with neutron number, i.e. a transition from prolate to oblate shape, but different models predict maximum triaxiality for different isotopes. The question whether the triaxial shape is rigid or $\gamma$-soft is not consistently described by theory.

It is difficult to measure the degree of triaxiality experimentally. Results from a multiple Coulomb excitation study of the heaviest stable isotope $^{104}$Ru were most consistent with triaxial deformation and a $\gamma$-soft potential [17, 18]. Results from a lifetime measurement of low-lying states in the ground-state and $\gamma$-band in $^{104}$Ru and $^{106}$Ru following inelastic scattering and two-neutron transfer, respectively, supported the notion of $\gamma$-softness in these nuclei [19]. Spectroscopic studies following the $\beta$ decay of Tc isotopes identified the band heads of the $K=2$ $\gamma$-bands in $^{106}$Ru and $^{108}$Ru [20] and in $^{110}$Ru and $^{112}$Ru [21]. The energy of the $2_2^+$ states is decreasing with neutron number (see Fig. 8), indicating increasing importance of triaxiality, and reaching a minimum for $^{112}$Ru [22]. The level schemes were extended to higher angular momentum by spectroscopy following spontaneous fission [23–25] and fusion-fission reactions [26, 27]. The characteristic odd-even energy staggering of the states in the $\gamma$ band suggests a transition from $\gamma$-soft excitations to more rigid rotation of the triaxial shape between $^{108}$Ru and $^{112}$Ru [24, 28]. Pairs of negative-parity bands in $^{110}$Ru and $^{112}$Ru have been interpreted as chiral vibrations, which can be uniquely associated with an underlying triaxial deformation [25]. An early onset of the band crossing for the alignment of $g_{9/2}$ protons in $^{111}$Ru has been interpreted as due to an increase in $\gamma$ deformation and a transition from prolate to oblate shape [27].

To gain more understanding of the nature and evolution of triaxility in the neutron-rich Ru isotopes, it is necessary to measure electromagnetic matrix elements. In the case of $^{110}$Ru, a low-energy Coulomb excitation experiment has yielded *B*(*E*2) values within the ground-state band up to the $6_1^+$ state, in-band and inter-band *B*(*E*2) values for the $\gamma$ band up to the $3_1^+$ state, and the spectroscopic quadrupole moment of the $2_1^+$ state [29]. The results are consistent with pronounced triaxial deformation near the ground state, and they are well reproduced both by the generalized triaxial rotor model and by beyond-mean-field calculations with the HFB gcm(goa) approach and the Gogny D1S interaction [29]. *B*(*E*2) values for in-band transitions at higher angular momentum within both the ground-state band and the $\gamma$ band in $^{110}$Ru were obtained from lifetime measurements using the Doppler shift attenuation method [30]. However, no experimental information on transition probabilities exists for the intermediate angular momentum range between $I=3$ and $I=6$ in $^{110}$Ru. This angular momentum range is particularly important for the understanding of triaxiality because most inter-band transitions from the $\gamma$ band to the ground-state band occur in this range. In the case of $^{112}$Ru, only the lifetime of the $2_1^+$ state [31] and a few in-band transition strengths at higher angular momentum [32] are known. Only longer-lived lifetimes in the nanosecond range are known for some states in $^{108}$Ru and the odd-mass isotopes from direct measurements on chemically separated Tc isotopes [33].

*B*(*E*2) values for transitions that are crucial for a quantitative understanding of triaxiality in the neutron-rich Ru isotopes are accessible through lifetime measurements of excited states in the picosecond range. Here we report on the measurement of 16 new lifetimes and 29 new *B*(*E*2) values for Ru isotopes with masses between $A=108$ and $A=112$, which were obtained in the same experiment using the same experimental recoil distance Doppler shift technique. The experimental setup and data analysis are described in Sect. 2. The results are presented in Sect. 3, followed by a discussion and comparison to theoretical models in Sect. 4. A summary and conclusion are presented in Sect. 5.

## Methods

### Experimental setup

A beam of  $~^{238}$U was accelerated by the ganil css1 cyclotron to 6.2 MeV/u and impinged on a $^{9}$Be target of areal density 1.85 mg $\hbox {cm}^{-2}$ to produce the nuclei of interest by fusion-fission reactions. The target was mounted in the Orsay Universal Plunger System (oups) [34], along with a $^{nat}$Mg degrader foil with areal density 4.5 mg $\hbox {cm}^{-2}$. The degrader served to slow down the decaying fission fragments, inducing a change in the Doppler shift that allowed distinguishing $\gamma$ rays emitted before or after the degrader. The thickness of the degrader was chosen to produce good separation between the two components of $\gamma$-ray transitions, while also ensuring that the fission products had high enough energy to be separated and identified in the Variable Mode Spectrometer (vamos++) [35]. Unreacted beam exited the target with an energy of approximately 5.3 MeV/u, which is well below the Coulomb barrier for Mg and hence low enough to only induce a negligible amount of sub-barrier fission on the degrader.

The Advanced Gamma Tracking Array (agata) [36] was used to detect the $\gamma$ decay of the fission fragments with high resolution. Covering a solid angle of about $1\pi$ between 175$^\circ$ and 135$^\circ$ from the vamos++ axis, the 41 high-purity germanium crystals were positioned to detect the $\gamma$ rays at angles where the Doppler shift is largest. Each crystal is 36-fold segmented, providing precise information on the location of $\gamma$-ray interactions in the crystal through pulse-shape analysis [37]. A tracking algorithm then reconstructs Compton scattering events, strongly reducing the Compton background [38, 39].

The magnetic ray-tracing spectrometer vamos++ was used to identify the fission fragments event by event. To optimize the transport of fragments of mass $A\approx 110$ to the focal plane, the spectrometer and the target chamber were rotated 19$^\circ$ with respect to the incoming beam, and the magnetic rigidity set to 1.11 T m. Position, angle and time were measured at the entrance of vamos++ [40] and in the focal plane, allowing for the reconstruction of particle trajectories and time of flight – and hence velocities – through the spectrometer event by event [35]. The fission fragments came to rest in a 6 by 5 pad ionization chamber at the focal plane, enabling a $\varDelta E$-*E* measurement of the nuclear charge. Further details about the agata and vamos++ setup can be found in Refs. [35, 41], and an overview of its performance in Ref. [42].

**Fig. 1:**
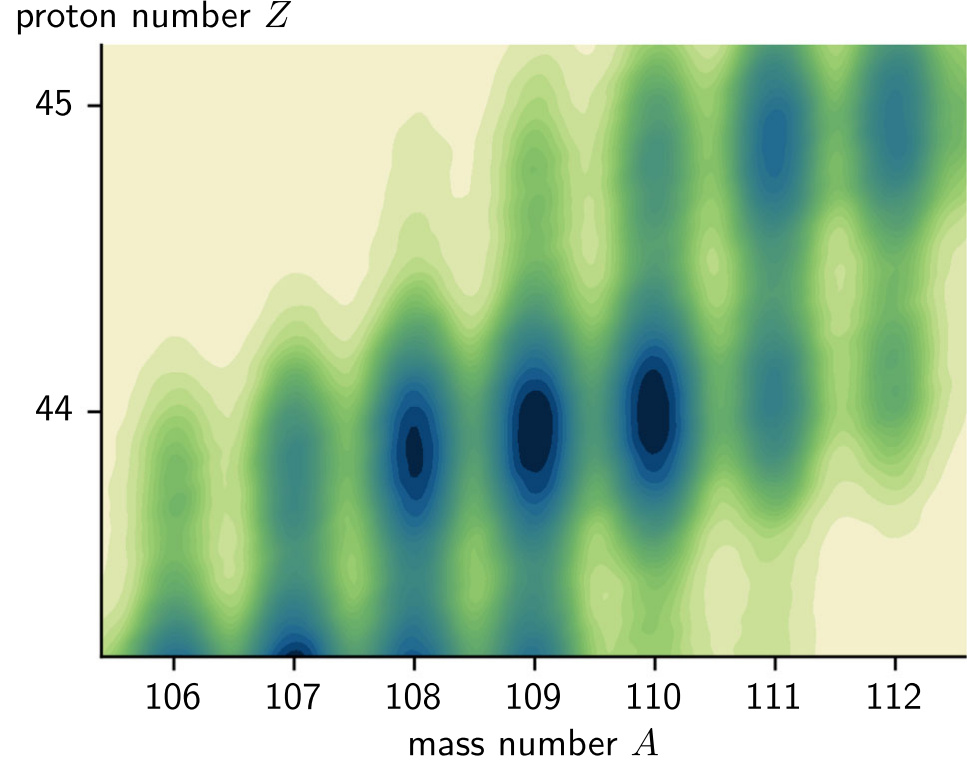
vamos++ particle identification density plot for the ruthenium chain ($Z = 44$)

We reconstructed the velocity of the ions before the degrader event by event, using the information from both the $\gamma$-ray energy measured in agata and the extracted charge, mass, position and angles in the vamos++ focal plane. The reconstructed velocities are shown in Table 1. About 100 different nuclides were identified in the experiment, although not all with enough statistics to measure lifetimes of excited states. The full chart of identified nuclides from this experiment was shown in a previous publication [43]. In this work we focus on the ruthenium chain, shown in Fig. 1.

Lifetimes of excited states in the picosecond range were measured with the recoil distance Doppler shift (rdds) method [44]. Throughout the experiment, oups measured the target–degrader distance by means of the capacitance between the two foils. Measurements were performed for ten distances with a measuring time of about 24 h per distance. The distances are reported in Table 2. We used these distances in conjunction with the velocity reconstructed from vamos++ and agata to determine the time at which the fragments reached the degrader.

**Table 1:** Velocity distributions of the fission fragments before (fast) and after (slow) the degrader. The values are based on the average $\langle 1/\beta \rangle$ instead of $1/\langle \beta \rangle$ following the recommendation of a previous study [45]. The standard deviations of the distributions are given in parentheses

Nucleus	$\langle 1/\beta \rangle ^{-1}_\textrm{slow}$	$\langle 1/\beta \rangle ^{-1}_\textrm{fast}$	$\beta$ distributions
$^{108}$Ru	0.106(6)	0.124(6)	
$^{109}$Ru	0.105(7)	0.123(6)	
$^{110}$Ru	0.104(7)	0.122(6)	
$^{111}$Ru	0.103(7)	0.121(6)	
$^{112}$Ru	0.103(7)	0.120(6)	

### Analysis

The $\gamma$ rays detected in agata were correlated in time with specific nuclides identified in vamos++. We then corrected the $\gamma$ ray energies for Doppler shifts using the velocities measured after the degrader. We produced $\gamma$ ray spectra and $\gamma$ $\gamma$ coincidence matrices for each nuclide and at each target–degrader distance.

States with half lives in the range of the flight time between target and degrader (about 1 ps to 70 ps, depending on the chosen distance) decay in comparable parts before and after the degrader. Because of the change in velocity, these fast and slow parts of the decay can be distinguished by different Doppler shift in the $\gamma$ ray spectra. With agata placed at backwards angles, the fast component will appear with a lower energy than the slow component, as seen in e.g. Fig. 4. For the shortest lifetimes, the rdds method is limited by cases where the decay during deceleration within the degrader becomes significant. This effect can be used to measure lifetimes around 1 ps and will be discussed in a forthcoming article. In the present study, which focuses on longer lifetimes, the effect is negligible compared to other uncertainties.

The extraction of lifetimes from rdds data relies on accurately determining the intensities of the slow and fast components, a task that can be rendered difficult by overlapping peaks and an intricate background. The main contributions to the background come from the decay of the complementary fission fragments produced in the same event, from the radioactive decay of fission fragments that were scattered into the chamber, and from remaining Compton background that was not removed by $\gamma$ ray tracking. Since the velocity vectors of the fission fragments that were not detected in vamos++ differ from those of the identified fragments, the energy distribution of the $\gamma$ rays is typically Doppler broadened enough that the total background is approximately linear over short energy intervals. Both components of the observed $\gamma$-ray transitions are to good approximation Gaussian. We binned the data with a width of 0.5 keV to improve computational efficiency, and performed maximum likelihood fits using the migrad algorithm [46] implemented in iminuit [47]. To ensure consistency between the fits at different target–degrader distances, we fitted all spectra simultaneously, using the same peak centroids and widths for all spectra.

**Table 2:** Summary of target–degrader distances

	Distance/$\upmu \hbox {m}$
d1	29.8(5)
d2	51.6(3)
d3	89.9(6)
d4	155(2)
d5	264(5)
d6	449(4)
d7	776(40)
d8	1170(5)
d9	1776(5)
d10	2650(5)

For cases with sufficiently high statistics, $\gamma$ $\gamma$ coincidence analysis is the most accurate way to determine lifetimes from rdds data. By requiring that a decay $i\rightarrow j$ be in coincidence with the fast component of a feeding transition $h\rightarrow i$, any influence from feeding from other states is removed along with much of the background. Let $I^\textrm{fast;slow}_{h\rightarrow i;i\rightarrow j}$ denote the intensity of the slow component of a transition $i\rightarrow j$ in coincidence with the fast component of a transition $h\rightarrow i$. We then obtain the lifetime through [44]

1 $$\begin{aligned} \tau _i \frac{\textrm{d}}{\textrm{d}t}{I^\textrm{fast;fast}_{h\rightarrow i;i\rightarrow j}(t)} = I^\textrm{fast;slow}_{h\rightarrow i;i\rightarrow j}(t). \end{aligned}$$

**Fig. 2:**
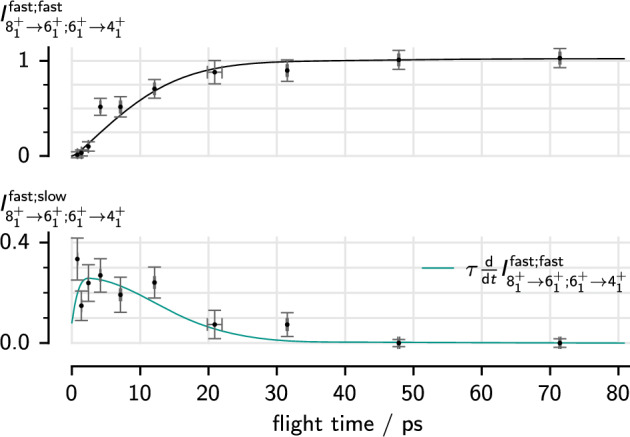
Fit of the lifetime to the decay curves of the $6^+_1\rightarrow 4^+_1$ transition in coincidence with the fast component of the $8^+_1\rightarrow 6^+_1$ transition in $^{108}$Ru. Estimated standard errors for the intensities and flight times are shown. The decay curve of the fast component and its derivative are fitted simultaneously to the data points of the fast and slow components, respectively, with the lifetime of the $6^+$ state as a scaling factor

We fit the intensities of both components of the gated peak simultaneously to this lifetime relation, using the lifetime itself as a fit parameter, as exemplified in Fig. 2. To correct for the difference in statistics between distances, we scale the intensities by that of another transition, typically the cleanest in the spectra to minimize the added uncertainty, that is the $2^+_1\rightarrow 0^+_1$ transition in the even isotopes and the $7/2^+_1\rightarrow 5/2^+_1$ transition in the odd isotopes.

**Fig. 3:**
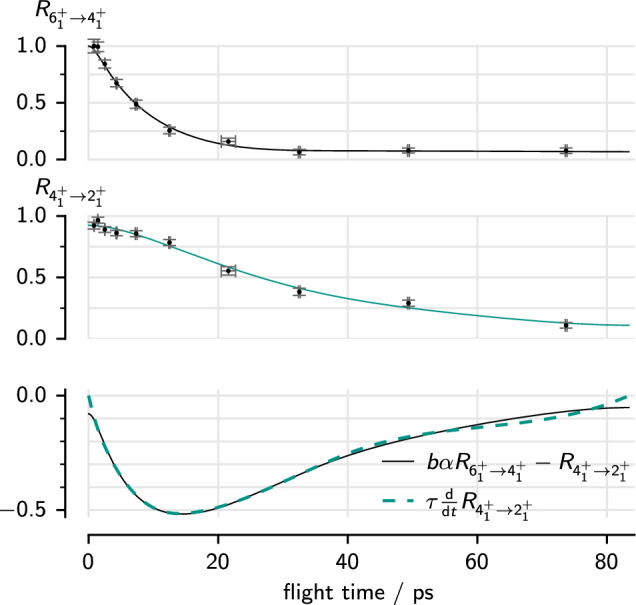
Fit of the lifetime to the decay curves of the $6^+_1\rightarrow 4^+_1$ transition and $4^+_1\rightarrow 2^+_1$ transition in $^{112}$Ru in $\gamma$-single analysis. Estimated standard errors on the relative intensities and flight times are shown. In addition to the data points, the two decay curves are fitted to the two sides of equation 2, shown in the bottom plot, with the lifetime of the $4^+_1$ state as a scaling factor

Single $\gamma$-ray analysis requires less statistics, but at the cost of adding systematic uncertainty. Let $R_{i\rightarrow j} = I^\textrm{slow}_{i\rightarrow j} / \left( I^\textrm{slow}_{i\rightarrow j} + I^\textrm{fast}_{i\rightarrow j}\right)$ be the relative intensity of the slow component of a transition $i\rightarrow j$. Then the lifetime is given by the relation [44]

2 $$\begin{aligned} \tau _i\frac{\textrm{d}}{\textrm{d}t}{R_{i\rightarrow j}(t)} = \sum _h b_{i\rightarrow j}\alpha _{h\rightarrow i} R_{h\rightarrow i}(t) - R_{i\rightarrow j}(t), \end{aligned}$$

where $b_{i\rightarrow j}$ is the branching ratio of the decay transition $i \rightarrow j$ and $\alpha _{h\rightarrow i}$ is a coefficient accounting for the fraction of the feeding that is observed from states *h*, corrected for the detector efficiency. We fit the decay and feeding curves simultaneously to lifetime relation 2, again using the lifetime itself as a fit parameter. An example is shown in Fig. 3.

All feeding transitions need to be accounted for in equation 2, but some might be too weak to measure. Unobserved feeding from a long-lived state could impact the lifetime result even for weak feeding transitions. However, the present study concerns states within rotational bands, where most of the feeding is observed. Unobserved feeding likely originates from the statistical decay of high-lying states populated in the fusion-fission reaction, which can be considered prompt and does not affect the measured lifetimes. All unobserved feeding is therefore considered prompt in the single $\gamma$-ray analysis. To avoid remaining uncertainties related to feeding, or overlapping peaks, we preferred a $\gamma$ $\gamma$ coincidence analysis where possible. Reference [43] provides a more detailed comparison and discussion of single $\gamma$ and $\gamma$ $\gamma$ coincidence analysis for this experiment.

Since equations 1 and 2 require the derivative of the decay curves, the curves need to be fitted with a differentiable function. An analytical model of the successive decay through several higher lying states would require too many free parameters to fit reliably. Nevertheless, these processes place some physical constraints on the decay curves which we can incorporate into our fit. Firstly, the decay curves must be sufficiently differentiable: at least the second derivative must be continuous. Secondly, the slow component – representing the nuclei which have yet to decay – must be monotonically decreasing, and the fast component must be increasing correspondingly. In the case of gating on the fast component of a feeder, the coincident intensity goes to zero at short flight times, and so does the slow component of the decay (see Fig. 2). We implemented these constraints by fitting the decay curves as B-splines [48]. These are $(k-1)$ times differentiable at order *k* and are easily constrained to be strictly decreasing or increasing. We chose the spline order for each curve to get the best fit, while avoiding overfitting. Figures 2 and 3 show examples of the fitting procedure for both $\gamma$ $\gamma$-coincidence and single $\gamma$ analysis. We follow the conclusion of a previous study [45] in using the mean of the inverse velocity $\langle 1/v\rangle$ to obtain the flight time used in the lifetime relations 1 and 2.

**Fig. 4:**
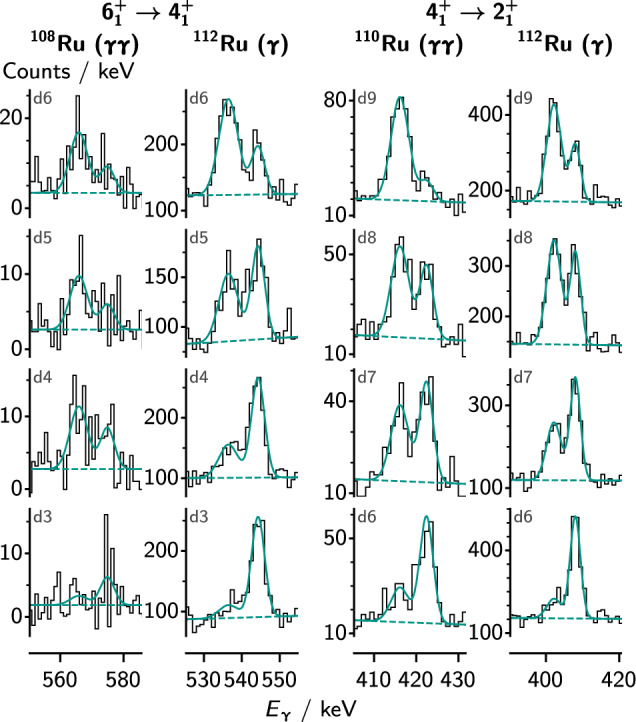
Spectra for transitions in the ground-state band in the even-even Ru isotopes, along with fits of the peaks. The spectra are Doppler corrected using the velocity after the degrader. The higher energy peak (slow component) then appears at the correct transition energy, whereas the fast component is shifted to lower energies. The labels indicate the target–degrader distances as presented in Table 2 and whether the spectra are $\gamma$-singles or $\gamma$ $\gamma$ coincidences. The data have been rebinned for visualization

**Fig. 5:**
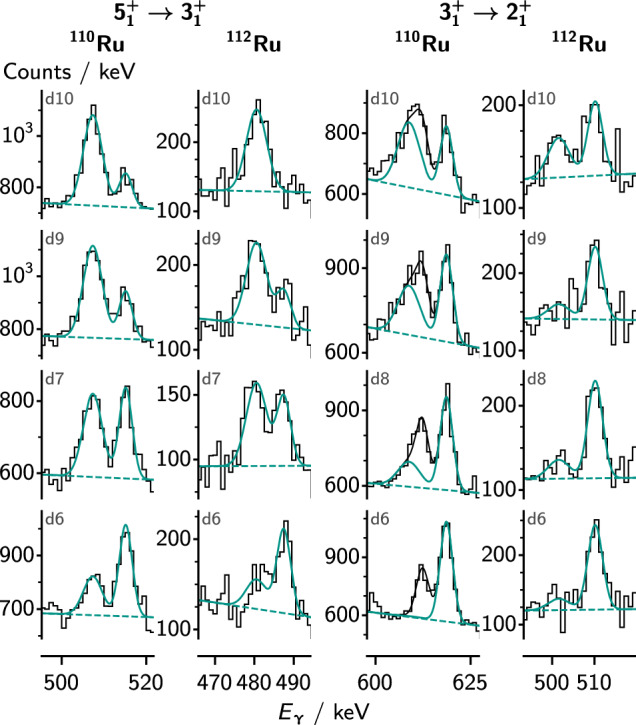
Single-$\gamma$ spectra for transitions within the $\gamma$ band in the even-even isotopes, Doppler corrected as in Fig. 4, along with fits of the peaks. The labels indicate the target–degrader distances as presented in Table 2. For the $3^+_1\rightarrow 2^+_1$ transition in $^{110}$Ru, the total fit includes the peak from the $2^+_2\rightarrow 0^+_1$ transition at 613 keV, which is partly overlapping with the fast component of the $3_1^+ \rightarrow 2_1^+$ transition. Because of the relatively long lifetime of the $2_2^+$ state, only the slow component of the $2_2^+ \rightarrow 0_1^+$ transition is visible. The data have been rebinned for visualization

**Fig. 6:**
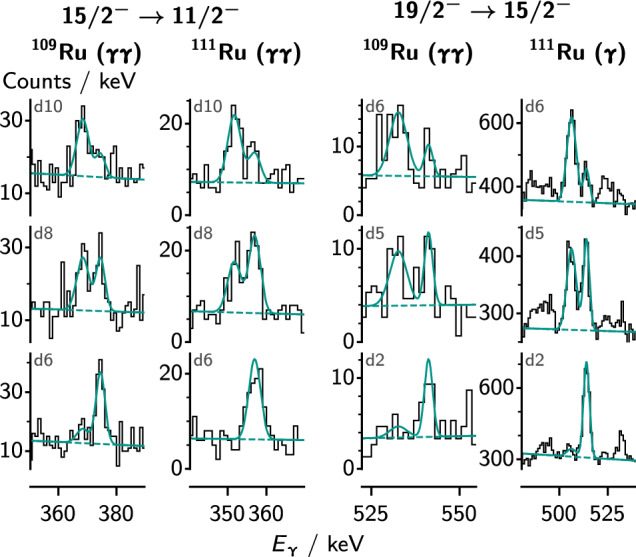
Spectra for transitions in the negative-parity bands of the odd-mass isotopes $^{109}$Ru and $^{111}$Ru, Doppler corrected as in Fig. 4, along with the fits of the peaks. The labels indicate the target–degrader distances as presented in Table 2 and whether the spectra are $\gamma$-singles or $\gamma$ $\gamma$ coincidences. The data have been rebinned for visualization

*B*(*E*2) values can be deduced from lifetimes assuming a known contribution of *E*2 $\gamma$-transitions to the overall decay of the state. We obtained internal conversion coefficients $\alpha _\textrm{IC}$ from BrIcc [49], but needed to make an assumption on the *E*2/*M*1 mixing ratios for some of the studied transitions. Experimental measurements of the $2^+_2\rightarrow 2^+_1$ and $3^+_1\rightarrow 2^+_1$ transitions have shown *M*1 contributions to be small in $^{108}$Ru [20]. Angular correlation measurements for the same transitions in $^{108}$Ru, $^{110}$Ru, and $^{112}$Ru, as well as the $5^+_2\rightarrow 4^+_1$ transition in $^{110}$Ru, also found very large *E*2/*M*1 mixing ratios, pointing to mostly pure *E*2 transitions [50]. For the other transitions with $\varDelta I < 2$, the mixing ratios are either unknown or not known with sufficient precision to be used in deriving *B*(*E*2) values. In light of this, we have chosen to assume pure *E*2 transitions for the $\gamma$ decay in all cases. Should this assumption be inaccurate, the true *B*(*E*2) value will be lower.

## Results

Figures 4, 5 and 6 show selected fits of the $\gamma$-ray spectra for the ground-state band and $\gamma$ band in the even-mass isotopes, and for the odd-mass isotopes, respectively. The figures indicate whether the spectra were obtained in single-$\gamma$ or $\gamma$ $\gamma$-coincidence analysis with a gate on the fast component of the in-band transition directly feeding the state of interest. Note that spectra are only shown for a selection of target-degrader distances, but that the data from all ten distances were used to determine the lifetimes. Partial level schemes of the even isotopes are shown in Fig. 8, and the lifetimes and *B*(*E*2) values are reported in Table 3. The *B*(*E*2) values of the transitions within the ground state bands of the even-even isotopes were presented previously [51] and are included for completeness.

**Fig. 7:**
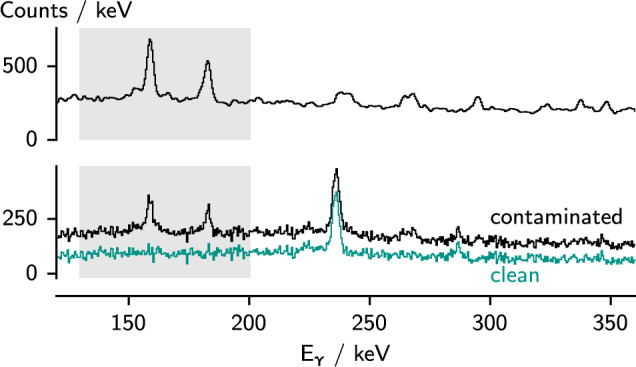
Top: smoothed $\gamma$ spectrum correlated with fragments identified as $^{112}$Rh. Bottom: $\gamma$ spectrum correlated with fragments identified as $^{112}$Ru before and after subtraction of the $^{112}$Rh contaminant. The scaling factor for the subtraction was chosen such that the contaminant peaks within the marked region were completely removed

**Fig. 8:**
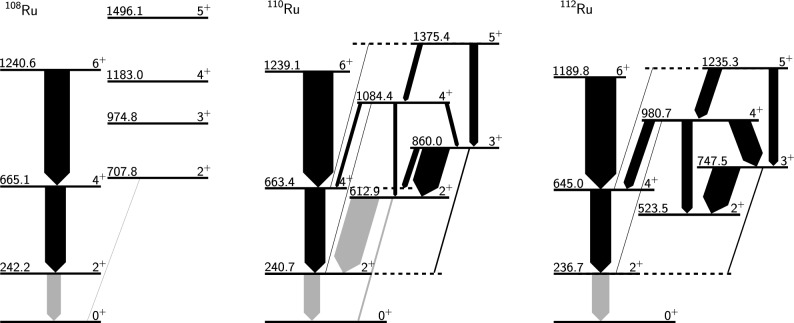
Partial level schemes for $^{108}$Ru, $^{110}$Ru and $^{112}$Ru. The level energies are given in keV, and the thickness of the arrows indicates the *B*(*E*2) values of the transition (see Table 3). Note the lowering of the $\gamma$ band with increasing neutron number. *B*(*E*2) values presented in this work are shown as black arrows, and the others (in gray) are taken from Refs. [29, 52, 56]

In $^{108}$Ru the $\gamma$ band lies higher in energy and is as such less populated in the fission reaction compared to the more neutron rich isotopes. Transitions from states in the $\gamma$ band of $^{108}$Ru were therefore too weak to extract any lifetimes; only transitions within the ground-state band could be analysed. The lifetime of the $2^+_1$ state is known to be (0.52 ± 0.04) ns [52], outside the sensitivity of this experiment. We obtained lifetimes for the $4^+_1$ and $6_1$ states with $\gamma$ $\gamma$ coincidence analysis. The lifetime of the $4^+_1$ state has only been measured as an average of the $4^+_1$ states in $^{108}$Ru and $^{110}$Ru, which could not be separated experimentally in a previous experiment [53]. There were indications of some remaining background under the $6^+_1$ state, which we subtracted using a gate on the region from 659.0 keV to 677.5 keV, in which no transitions were present.

$^{110}$Ru is the ruthenium isotope with the highest statistics in our dataset. In the $\gamma$ band, we obtained lifetimes for the $3^+_1$, $4^+_2$, and $5_1^+$ states (see Fig. 5). The fast component of the $3^+_1\rightarrow 2^+_1$ transition overlaps with the slow component of the $2^+_2\rightarrow 0^+_1$ transition. Since the branching ratio for the decay of the $2_2^+$ state is known [54], we were able to account for the $2_2^+ \rightarrow 0_1^+$ transition by simultaneously fitting the $2^+_2\rightarrow 2^+_1$ transition and thus constrain not only the energy and width, but also the intensity of the $2_2^+ \rightarrow 0_1^+$ transition in the fit. In the ground state band, we obtained lifetimes for the $4^+_1$ and $6^+_1$ states – in $\gamma$ $\gamma$-coincidence analysis for the first and in both $\gamma$ $\gamma$-coincidence and single-$\gamma$ analyses for the second. The difference between the two techniques was not statistically significant in this case. The lifetimes in the ground state band have both been measured previously [55] and our values are consistent.

**Table 3:** Lifetimes and *B*(*E*2) values from the present work. The *B*(*E*2) values are deduced assuming pure *E*2 transitions. The errors on the *B*(*E*2) values indicate estimates of the 16th and 84th percentile given this assumption

$I_\textrm{i}$	lifetime / ps		$I_\textrm{f}$	$E_\gamma$ / keV	*b* / %	$\alpha _\textrm{IC}$ / ${10^{-3}}$	$B(E2;\downarrow )$ / ${\hbox {e}^{2}\hbox {b}^2}$
	This work	Literature					
— $\boldsymbol{^{109}}$**Ru** —							
$15/2^{-}_{1}$	^c^45(6)		$11/2^{-}_{1}$	374.7	100.0^*^	12.95	$0.242^{+0.037}_{-0.028}$
$19/2^{-}_{1}$	^c^4.8(10)		$15/2^{-}_{1}$	541.6	100.0^*^	4.24	$0.36^{+0.10}_{-0.06}$
— $\boldsymbol{^{111}}$**Ru** —							
$15/2^{-}_{1}$	^c^57(10)		$11/2^{-}_{1}$	357.8	100.0^*^	15.02	$0.24^{+0.05}_{-0.04}$
$19/2^{-}_{1}$	^s^4.9(9)		$15/2^{-}_{1}$	514.1	100.0^*^	4.92	$0.46^{+0.10}_{-0.07}$
— $\boldsymbol{^{108}}$**Ru** —							
$4^+_1$	^c^22.3(23)		$2^+_1$	422.9	100.0	8.85	$0.268^{+0.031}_{-0.025}$
$6^+_1$	^c^3.9(9)		$4^+_1$	575.5	100.0	3.57	$0.33^{+0.10}_{-0.06}$
— $\boldsymbol{^{110}}$**Ru** —							
$3^+_1$	^s^42(5)	$48.8^{+0.7}_{-0.6}$^a^	$2^+_1$	619.2	81.8(14)	2.92	^e^$0.0174^{+0.0024}_{-0.0019}$
			$2^+_2$	247.1	16.9(14)	51.9	^b^$0.34^{+0.06}_{-0.05}$
			$4^+_1$	196.6	1.25(17)	115.6	^b^$0.074^{+0.015}_{-0.012}$
$4^+_1$	^c^22.6(21)	15.4(17) [55], $20.6^{+1.6}_{-2.0}$^a^	$2^+_1$	422.6	100.0	8.87	$0.266^{+0.027}_{-0.023}$
$4^+_2$	^s^33(4)		$2^+_2$	471.5	46(4)	6.36	$0.049^{+0.008}_{-0.007}$
			$2^+_1$	843.6	28.8(32)	1.317	$0.00167^{+0.000 30}_{-0.000 25}$
			$4^+_1$	421.0	23.5(17)	8.98	^b^$0.044^{+0.007}_{-0.006}$
			$3^+_1$	224.5	1.25(12)	72.5	^b^$0.051^{+0.009}_{-0.007}$
$5^+_1$	^s^16.1(31)		$3^+_1$	515.5	80.7(4)	4.89	$0.112^{+0.027}_{-0.018}$
			$4^+_1$	711.9	16.4(4)	2.014	^e^$0.0045^{+0.0011}_{-0.0007}$
			$4^+_2$	291.0	2.9(15)	29.7	^b^$0.07\pm 0.04$
$6^+_1$	^s^3.3(5)	2.4(10) [55], $3.40^{+0.40}_{-0.65}$^a^	$4^+_1$	575.7	100.0	3.56	$0.39^{+0.07}_{-0.05}$
$6^+_1$	^c^4.0(8)		$4^+_1$	575.7	100.0	3.56	$0.32^{+0.08}_{-0.05}$
— $\boldsymbol{^{112}}$**Ru** —							
$3^+_1$	^s^112(26)		$2^+_1$	510.8	72(4)	5.02	^e^$0.0150^{+0.0046}_{-0.0029}$
			$2^+_2$	224.0	28(4)	73	^b^$0.34^{+0.12}_{-0.08}$
$4^+_1$	^s^26.4(24)		$2^+_1$	408.2	100.0	9.88	$0.270^{+0.027}_{-0.023}$
$4^+_2$	^s^22(10)		$2^+_2$	457.2	75(5)	6.97	$0.14^{+0.12}_{-0.04}$
			$4^+_1$	335.6	14.9(34)	18.5	^b^$0.13^{+0.11}_{-0.05}$
			$3^+_1$	233.2	5.3(13)	63.5	^b^$0.27^{+0.24}_{-0.10}$
			$2^+_1$	744.0	5.3(13)	1.798	$0.0009^{+0.0008}_{-0.0003}$
$5^+_1$	^s^21.2(28)		$3^+_1$	487.9	88(11)	5.74	$0.122^{+0.025}_{-0.020}$
			$4^+_1$	590.3	7.1(12)	3.32	^b^$0.0038^{+0.0009}_{-0.0008}$
			$4^+_2$	254.7	5.01(22)	46.8	^b^$0.172^{+0.027}_{-0.021}$
$6^+_1$	^s^4.2(8)		$4^+_1$	544.9	100.0	4.16	$0.40^{+0.09}_{-0.06}$

$^\text {s}$single-$\gamma$  $^\text {c}\gamma \gamma$-coincidences  $^\text {a}$Derived from Coulomb excitation measurement [29]

$^\text {b}$Assuming pure *E*2 transition.  $^\text {e}$Experimentally negligible *M*1 [50].

$^\text {*}$Neglecting unobserved low energy transition. See text for details

$^{112}$Ru, unlike the other isotopes, showed clear contamination from the isobar $^{112}$Rh. The overlap with $^{112}$Rh, which is produced with higher yield in the fission reaction, can be seen in Fig. 1. We used a procedure to subtract $\gamma$-ray spectra correlated with ions identified as $^{112}$Rh from those correlated with $^{112}$Ru. The energy range from 130 keV to 200 keV contains two strong transitions in $^{112}$Rh, but none in $^{112}$Ru, and can therefore be used to quantify the amount of contamination from $^{112}$Rh. We smoothed the contaminating $^{112}$Rh spectra by convolving them with a Gaussian kernel with standard deviation 1 keV, and subtracted the result from the $^{112}$Ru spectra with a scaling such that the peaks in the aforementioned energy region were completely removed. An example is shown in Fig. 7. The resulting spectra did not have enough statistics to perform a $\gamma$ $\gamma$ coincidence analysis, but we extracted several lifetimes in single $\gamma$ analysis. In the $\gamma$ band, we obtained the lifetime of the $3^+_1$, $4^+_2$ and $5^+_1$ states. In the ground state band, we obtained the lifetimes of the $4^+_1$ and $6^+_1$ states. The $3^+_1\rightarrow 2^+_1$ transition at 511 keV, the $2^+_2\rightarrow 0^+_1$ transition at 523 keV, and the $6_1^+ \rightarrow 4_1^+$ transition at 545 keV are relatively close in energy, making it difficult to define a clear background region. To get a good background estimate, we fitted the $3^+_1 \rightarrow 2^+_1$ and $6_1^+ \rightarrow 4_1^+$ transitions simultaneously within the same energy interval, excluding the long-lived $2_2^+ \rightarrow 0_1^+$ transition from the fit. For each transition, only the primary feeder was present in the spectra, and could thus be taken into account. Concerning the $4^+_1$ state, it is known that a weak $4^+_2\rightarrow 4^+_1$ transition contributes to its feeding along with the main feeding from the $6_1^+$ state. However, given that the strength of the $4_2^+ \rightarrow 4_1^+$ is only 20% of the weakly observed $4^+_2\rightarrow 2^+_2$ transition [56], the former can be safely neglected. The fits of the $6^+_2\rightarrow 4^+_2$ and $4^+_2\rightarrow 2^+_2$ transitions were hampered by remaining $^{112}$Rh contamination and other structure in the background, which resulted in a large uncertainty in the lifetime of the $4^+_2$ state.

In the odd mass ruthenium isotopes, only the negative-parity yrast band (shown in Fig. 13) was populated enough to extract lifetimes. For $^{109}$Ru, we obtained lifetimes of the $15/2^{-}_{1}$ and $19/2^{-}_{1}$ states, both in $\gamma \gamma$ coincidence analysis. For $^{111}$Ru, we obtained lifetimes of the $15/2^{-}_{1}$ state in $\gamma$ $\gamma$ coincidence analysis and of the $19/2^{-}_{1}$ state in single $\gamma$ analysis. Fits of the spectra are shown in Fig. 6.

**Fig. 9:**
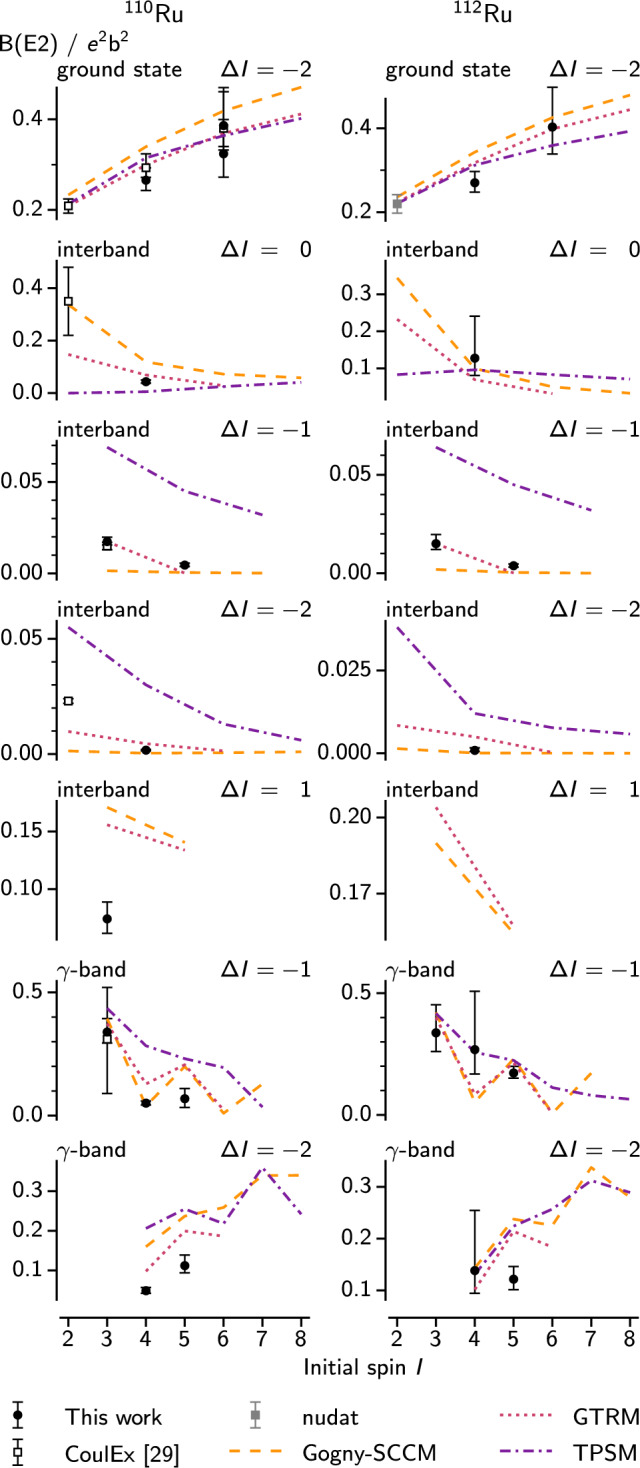
Experimental and predicted *B*(*E*2) values for $^{110}$Ru (left) and $^{112}$Ru (right). Data points from [29, 56] are included for comparison, as are tpsm calculations from [61]

## Discussion

The experimentally deduced *B*(*E*2) values are important to test theoretical calculations. In the even-even isotopes, fully microscopic models are possible, and we present new results from a state-of-the-art beyond mean field calculation. We also use our data to fit phenomenological models, from which we can extract the deformation of the nucleus under a set of assumptions. The even-even isotopes are well described by the generalized triaxial rotor model, whereas for the odd neutron isotopes we use the triaxial particle rotor model.

### Symmetry conserving configuration mixing

These calculations [57] implement the generator coordinate method [58] with the Gogny D1S effective interaction [59], using triaxial Hartree-Fock-Bogoliubov wave functions found with variation after particle number projection (vapnp) [60]. No fitted parameters – other than the global parameters of the Gogny interaction – are necessary. The results are shown in Fig. 9.

The predicted *B*(*E*2) values (shown with dashed orange lines in Fig. 9), agree remarkably well, overall, with the experimental data. While the calculations do not reproduce the observed decrease from $3^+_1\rightarrow 2^+_1$ to $5^+_1\rightarrow 4^+_1$, they do reproduce the decrease from $2^+_2\rightarrow 2^+_1$ to $4^+_2\rightarrow 4^+_1$ in $^{110}$Ru. In the $\gamma$ band, the model predicts a staggering of the *B*(*E*2) values with transitions from odd spin states being stronger than from even spin states. Unfortunately the uncertainties on our experimental values prevent us from drawing a definitive conclusion. The calculations find very small *M*1 contributions to the decays, consistent with our assumption of pure *E*2 transitions in the experimental data.

Results from the triaxial projected shell model (tpsm) calculations of [61] are included in Fig. 9 as purple dashed-dotted lines. While they agree well with the data in the ground state band, there is less agreement for the transitions from the $\gamma$ band to the ground state band, compared to the other models. The tpsm also predicts the staggering of *B*(*E*2) values in the $\gamma$ band, but to a lesser degree than the other models, except for the $\varDelta I = -2$ transitions in $^{110}$Ru.

**Fig. 10:**
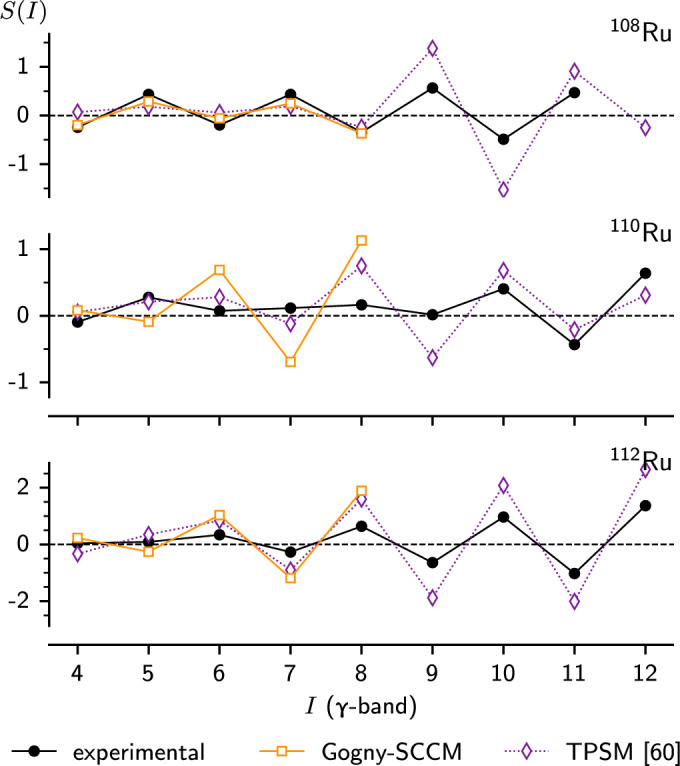
Staggering patterns calculated from equation 3 for $^{108}$Ru, $^{110}$Ru and $^{112}$Ru. The Gogny-sccm calculations reproduce the staggering flip in $^{110}$Ru, but at lower spin than the experimental values

**Fig. 11:**
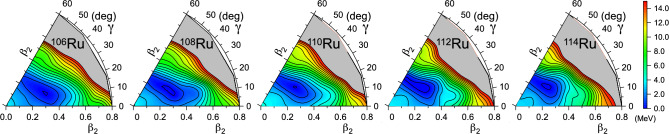
Particle-number projected energy surfaces in the $(\beta _{2},\gamma )$ plane computed with Gogny D1S energy density functional

The staggering of the odd and even energy levels in the $\gamma$ band

3 $$\begin{aligned} S(I) = \frac{[E(I)-E(I-1)]-[E(I-1)-E(I-2)]}{E(2^+_1)}, \end{aligned}$$

is an important property relating to the $\gamma$ rigidity of a deformed nucleus [62]. While the absolute magnitudes of the energy levels predicted by the Gogny-sccm calculations are 20%–60% too high, the model nevertheless succeeds in reproducing most features of the staggering pattern. As seen in Fig. 10, there is an inversion of the staggering between $^{108}$Ru and $^{112}$Ru, suggesting a transition from $\gamma$ soft to $\gamma$ rigid motion. A better quantitative agreement with the experimental absolute values is expected whenever time-reversal symmetry breaking intrinsic states, e.g. cranking HFB states, are included in the sccm calculation [63].

There is also an increase in staggering amplitude with spin, which the calculations reproduce. A discrepancy is still present in $^{110}$Ru, where the inversion occurs around $I=7$, but the calculations show inversion from the start ($I=4$). The tpsm calculations are somewhat closer to the data, producing the inversion around $I=5$. As pointed out in [61], a staggering in the *B*(*E*2) values within the $\gamma$ band with opposite phase of the energy staggering is expected for $\gamma$ rigid rotation. Such a staggering is also reproduced by the Gogny-sccm model, as seen in Fig. 9.

The potential energy surfaces from Gogny HFB calculations are shown in Fig. 11. All potential energy surfaces exhibit triaxial minima, with $\gamma$ deformation gradually increasing with neutron number, evolving from predominantly prolate configurations to minima near $\gamma =30^{\circ }$ in $^{110,112}$Ru, and $\gamma >30^{\circ }$ in $^{114}$Ru. Furthermore, $\gamma$-softness remains significant, although the surfaces become stiffer and tend toward oblate shapes in $^{112,114}$Ru.

### Generalized triaxial rotor model

The generalized triaxial rotor model (gtrm) [64] is a model of collectivity in even-even nuclei of a fixed triaxial deformation. It improves upon the triaxial rotor model proposed by Davydov and Filippov [65] by letting go of the assumption of irrotational flow moments of inertia, instead retaining them as free parameters. In the gtrm, *E*2 matrix elements are functions of the quadrupole moment $Q_0$, asymmetry angle $\gamma$ and mixing angle $\varGamma$ between the ground state band and $\gamma$-band. These parameters can be fitted analytically to experimental data.

We fitted the gtrm to *B*(*E*2) values for the $2^+_1\rightarrow 0^+_1$ and $3^+_1\rightarrow 2^+_1$ transitions, and to the branching ratio of the $\gamma$ band $2^+_2$ state to the $0^+_1$ and $2^+_1$ states. We then extrapolated the fit to predict transition strengths from higher-lying states.

Figure 9 shows that the gtrm largely succeeds in reproducing the magnitudes and trends of the experimental *B*(*E*2) values, also when extrapolated. In the ground state band, the *B*(*E*2) values are increasing with spin as expected from a rotational band, and are well reproduced by the model. Among the transitions from the $\gamma$ band to the ground state band, those without spin change are much stronger than the others. Curiously, the gtrm does not reproduce the higher value for $2^+_2\rightarrow 2^+_1$ in $^{110}$Ru, but does predict it in $^{112}$Ru, for which we lack that data point. In the $\gamma$ band, we see the same staggering pattern as in the Gogny-sccm calculations. This pattern is expected when the deformation is constant [61], as is assumed by the gtrm.

The full fit parameters for the gtrm are listed in Table 4. Previous measurements [29] established $^{110}$Ru as the strongest case for triaxiality near the ground state, with $\gamma =({29.0 \pm 4.8})^\circ$. The results presented here place $^{112}$Ru in this position with $\gamma =({26 \pm 4})^\circ$, while yielding a notably weaker triaxiality of $\gamma = ({22.6 \pm 0.9})^\circ$ for $^{110}$Ru. The discrepancy may be due to the empirical quantities used in the fit: the previous measurements relied on the quadrupole moment of the $2^+_1$ state, which had a significantly larger uncertainty than the quantities used here.

**Table 4:** Optimal fit parameters for the generalized triaxial rotor model

	$Q_0$ / e b	$\gamma$ / $^{\circ }$	$\varGamma$ / $^{\circ }$
$^{110}$Ru	3.32(11)	22.6(9)	-10.4(7)
$^{112}$Ru	3.42(16)	26(4)	-15(4)

**Fig. 12:**
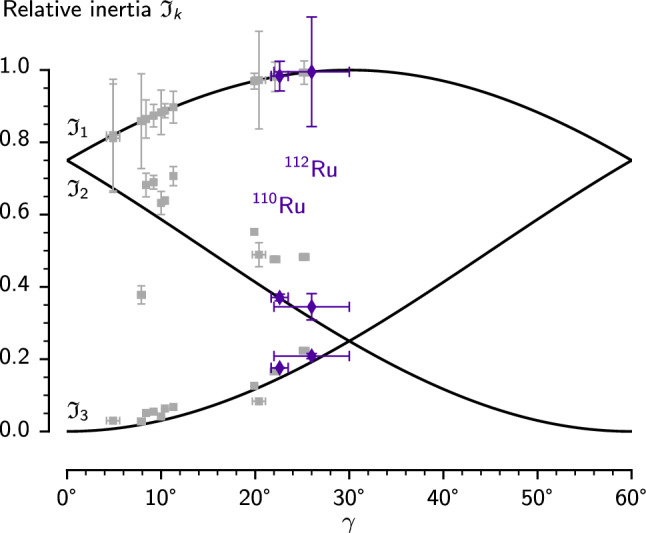
Relative moments of inertia for each axis of the gtrm. The values are scaled to the irrotational flow line through the 1-axis. The relative values from [66] show that triaxial nuclei tend to qualitatively follow the irrotational flow pattern. However, the magnitude of the moments of inertia are in between irrotational and rigid flow expectations for all nuclei. The $^{110}$Ru data point from [66] has been omitted from this figure and was replaced by the values from the present work

The gtrm also lets us investigate the moments of inertia themselves. A study of moments of inertia deduced from a dozen triaxial nuclei [66] found that the relative moments of inertia lie close to those expected for irrotational flow. This property has since been reproduced in both covariant density functional theory [67] and semiclassical cranked Hartree-Fock-Bogoliubov [68] calculations. As we see in Fig. 12, $^{110}$Ru and $^{112}$Ru fit neatly into this pattern. The fact that the mixing parameter $\varGamma$ is much larger for $^{112}$Ru than for $^{110}$Ru is consistent with the larger degree of triaxiality [66].

### Triaxial particle-rotor model

The triaxial particle-rotor model (tprm) is a collective model of the odd mass nucleus as an even-even triaxially deformed core coupled to a single odd particle [69]. The rotation of the core itself is assumed to be fully rigid, with a fixed deformation $(\epsilon _2, \gamma )$, and its moment of inertia determined by the energy of the first $2^+$ state^1^ and – if necessary – a coriolis attenuation factor $\xi$.

The deformed mean field of the core is parametrized with a modified oscillator potential. The neutron Nilsson states in this potential are then calculated [70], and a subset around the Fermi level selected for coupling to the odd neutron. The particle-rotor Hamiltonian is constructed in the strong coupling basis [71], with residual pairing treated within the bcs approximation with standard values for the pairing strength parameter and deriving the Fermi level and pairing gap [72]. The energy levels are then obtained by diagonalizing the Hamiltonian, and the eigenvectors are used to calculate the electromagnetic matrix elements and $\delta (E2/M1)$ mixing ratios. Following standard practice, the *g* factor of the core was approximated as $g_R = Z/A$, and the intrinsic $g_s$ factor as 65% of the free value.

**Table 5:** Optimal fit parameters for the triaxial particle rotor model

	$\epsilon _2$	$\gamma$	$E(2^+_1)_\textrm{core}$	$\xi$
			/ keV	
$^{109}$Ru	0.279	18.4$^\circ$	179	0.79
$^{111}$Ru	0.325	27.5$^\circ$	232	1.00

We used $^{108}$Ru and $^{110}$Ru cores to study $^{109}$Ru and $^{111}$Ru, respectively, and optimized the model parameters to reproduce the observed energy levels $\boldsymbol{E}_\textrm{obs}\pm \boldsymbol{s}_\textrm{obs}$ from [73, 74]. We defined the log-likelihood function

4 $$\begin{aligned} \ell (\boldsymbol{\theta }|\boldsymbol{E}_\textrm{obs}) = -\frac{1}{2}\sum _{i=1}^n \frac{\left[ E_{\textrm{obs}\,i} - f_i(\boldsymbol{\theta })\right] ^2}{s_{\textrm{obs}\,i}^2} + \ln (s_{\textrm{obs}\,i}^2), \end{aligned}$$

where $\boldsymbol{\theta }$ denotes the model parameters and $\boldsymbol{f}(\boldsymbol{\theta })$ the predicted energy levels. We sampled the posterior distribution of $\boldsymbol{\theta }$ using the Markov-chain Monte Carlo implementation emcee [75]. Following the recommendations given in [76, 77], we used a differential evolution proposal with 10% snooker updates and 90% parallel direction updates. These fits of the tprm find triaxiality close to the next even-even nucleus, with $\gamma ={18.4}^{\circ }$ for $^{109}$Ru and $\gamma ={27.5}^{\circ }$ for $^{111}$Ru. The full fit parameters are listed in Table 5.

**Fig. 13:**
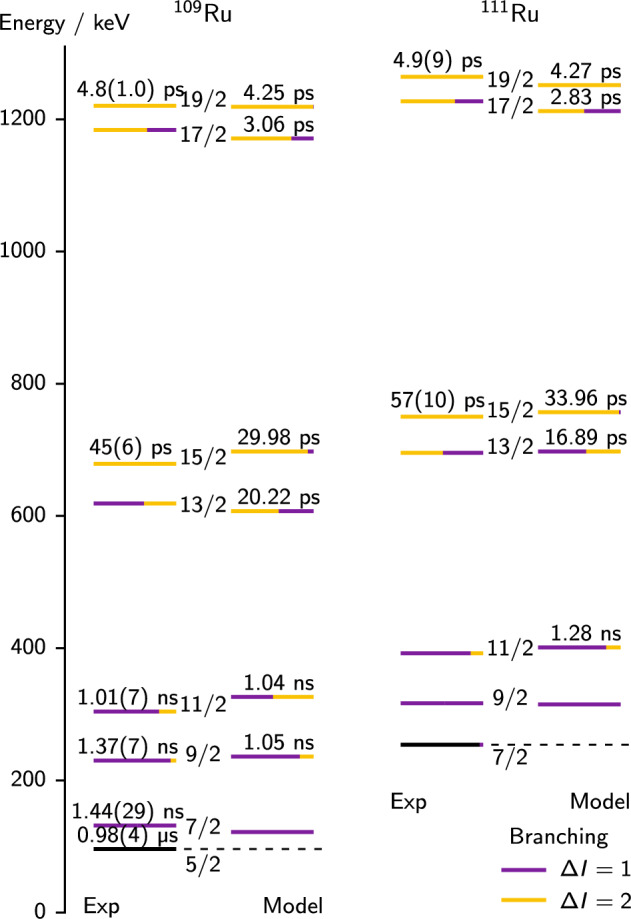
Results of the particle-rotor model compared to experimental data from this work and [73, 74]. The energies of the particle-rotor model are calculated relative to the lowest state, marked by a dashed line. The model lifetimes are obtained using experimental energies (see text for details). Colour indicates the branching ratios

The tprm reproduces the experimental energies remarkably well as seen in Fig. 13. While the predicted and measured branching ratios are quite close in many cases, the difference still affects the *B*(*E*2) values, especially for the $15/2^{-}$ states, for which the transitions to the $13/2^{-}$ states have not been measured, and are therefore assumed zero in Table 3. Because of this, Fig. 13 compares experimental lifetimes with lifetimes obtained from the tprm *B*(*E*2) values and branching ratios, using the experimental transition energies and internal conversion corrections from BrIcc. We show predicted *B*(*E*2) and *B*(*M*1) values in Table 6. The predicted lifetimes are consistent with experiment in several cases, but a few differ considerably. The model especially underestimates the lifetime of the $15/2^{-}$ state, hence overestimating the *B*(*E*2) value.

**Table 6:** Reduced transition probabilities predicted by the triaxial particle-rotor model compared with our values. Note that the latter are neglecting the unobserved $\varDelta I = 1$ transitions

	$I_\textrm{i}$	$I_\textrm{f}$	*B*(*M*1)	*B*(*E*2) / ${\hbox {e}^{2}\hbox {b}^{2}}$	
			${/{{\mu }{N}}^{2}}$	tprm	Exp.
$^{109}$Ru	$15/2^{-}_{1}$	$11/2^{-}_{1}$		0.3393	0.242(32)
		$13/2^{-}_{1}$	0.2563	0.1477	
	$19/2^{-}_{1}$	$15/2^{-}_{1}$		0.4093	0.36(8)
		$17/2^{-}_{1}$	0.3643	0.0911	
$^{111}$Ru	$15/2^{-}_{1}$	$11/2^{-}_{1}$		0.3962	0.24(4)
		$13/2^{-}_{1}$	0.2268	0.2093	
	$19/2^{-}_{1}$	$15/2^{-}_{1}$		0.5297	0.46(8)
		$17/2^{-}_{1}$	0.2939	0.1674	

The particle-rotor calculations indicate that the $[532]5/2^{-}$ Nilsson orbital of $h_{11/2}$ parentage dominates the wave functions of the states in the negative-parity band in $^{109}$Ru. With increasing angular momentum, the wave functions spread over several values of K, which is no longer a good quantum number because of the triaxiality. In addition, there is an increasing contribution from the $[541]3/2^{-}$ Nilsson orbital, although the $\varOmega =5/2$ and $K=5/2$ components remain dominant. In the case of $^{111}$Ru, the $K=7/2$ component of the $[523]7/2^{-}$ orbital is the main contribution in the wave functions of the negative-parity band. There is an increasing contribution from the $\varOmega =5/2$ orbital with angular momentum, reaching similar amplitudes to the $\varOmega =7/2$ contribution for the $19/2^{-}$ and $23/2^{-}$ states. The *K* mixing is even stronger for $^{111}$Ru than for $^{109}$Ru, reflecting the increase in triaxiality.

The decoupling of the negative-parity bands in the odd-mass Ru isotopes, leading to almost degenerate energy levels for states with angular momenta *I* and $(I+1)$, was discussed in previous works [78, 79]. Hwang et al. speculated that an admixture of the $[541]1/2^{-}$ orbital of $f_{7/2}$ and $h_{9/2}$ parentage could lead to a decoupling of the band [78], providing an alternative explanation to a rotation alignment by the Coriolis force. Our calculations find no indication of any contribution from the $[541]1/2^{-}$ orbital and support the interpretation of rotation alignment as the cause of the decoupling. For $^{111}$Ru with $\gamma =27.5^{\circ }$, the collective angular momentum in the $\alpha =+1/2$ branch of the band is almost fully aligned with the axis of largest moment of inertia (the 1-axis in Fig. 12), while some collective angular momentum is shifted to the axis with second largest moment of inertia (the 2-axis in Fig. 12) for the $\alpha =-1/2$ branch. The calculations find a similar shift in collective angular momentum from the 1-axis to the 2-axis for the $\alpha =-1/2$ branch in $^{109}$Ru, although there is generally more sharing of collective angular momentum between the two axes due to the smaller value of $\gamma =18.4^{\circ }$.

The mechanisms of rotation alignment and the spreading of the wave functions over different single-particle orbitals was in general discussed by Larsson et al. in the context of the particle-rotor model for bands based on $h_{11/2}$ intruder configurations [71]. There, it was shown that particle and hole states favour prolate and oblate deformation, respectively, whereas triaxial shapes are favoured in the middle of the shell. Our results indicate that the neutron-rich Ru isotopes exemplify these mechanisms, which explain the increase of $\gamma$ deformation with neutron number.

## Summary and conclusions

Using the agata coupled to vamos++ setup at ganil, we have measured 16 lifetimes with the recoil distance Doppler-shift method and obtained 29 *B*(*E*2) values in $^{108-112}$Ru. We compared these to both phenomenological and fully microscopic models, and found an overall good agreement. The models indicate that the degree of triaxiality is increasing with neutron number, reaching maximum triaxiality in $^{112}$Ru. In the even isotopes, the fully microscopic Gogny-sccm calculations point to a transition from predominantly $\gamma$ soft to more $\gamma$ rigid behaviour with increasing neutron number. The good qualitative agreement between the Gogny-sccm calculations and experimental *B*(*E*2) values supports this interpretation. A more quantitative agreement is expected if cranking-HFB wave functions are included within the sccm set. Such calculations are presently being developed. The fitted gtrm parameters show that the relative moments of inertia qualitatively follow an irrotational pattern, consistent with an increase in triaxiality for ruthenium and with the systematics of what has been observed for triaxial nuclei across a wider mass region. In the odd isotopes, the fitted tprm gives insight into the underlying configuration of the odd neutron. The alignment of angular momenta along the three axes is consistent with the moments of inertia obtained from the gtrm for the even-mass nuclei and explains the decoupling of the bands.

## Data Availability

This manuscript has associated data in a data repository. It may be made available upon reasonable request to the agata collaboration.

## Footnotes

1 Since the core is not actually a rigid rotor, this energy is usually somewhat lower than the energy of that state in the corresponding even-even nucleus.

## Code Availability Statement

The code associated with this manuscript will be made available upon reasonable request.
